# *WWOX*-related epileptic encephalopathy caused by a novel mutation in the *WWOX* gene: a case report

**DOI:** 10.3389/fped.2024.1453778

**Published:** 2024-10-02

**Authors:** Dan Feng, Ye Li, Ya-Ting Zhang, Yan-Jun Song, Dong-Yuan Qin, Fan Wang

**Affiliations:** ^1^ The Second Clinical Medical College of Lanzhou University, Lanzhou, China; ^2^ Department of Neonatology, Lanzhou University Second Hospital, Lanzhou, China

**Keywords:** epileptic encephalopathy, infantile epilepsy, *WWOX* gene, compound heterozygous mutations, whole-exome sequencing, WOREE syndrome

## Abstract

**Background:**

*WWOX*-related epileptic encephalopathy is an autosomal recessive disorder caused by mutations in the WW-containing oxidoreductase gene, characterized by the onset of refractory seizures in infants. Early-onset epilepsy, electroencephalography abnormalities, and developmental delay or degeneration are the main clinical manifestations. Early death can occur in severe cases. In the present study, a novel variant in *WWOX* was detected in a patient with epilepsy and his healthy parents.

**Case presentation:**

A 5-month-old boy presented with epilepsy. The main manifestations were intractable seizures, mental and motor retardation and hearing impairment. Subsequent genetic testing revealed the presence of an epilepsy-associated novel mutation: c.991C>A (amino acid change: p.Ser304Tyr) in the *WWOX* gene. Variants were inherited from parents with healthy phenotypes. Finally, a patient died at 6 months of age.

**Conclusion:**

The discovery of novel variants has enriched the existing database of *WWOX* gene variants and may expand the range of clinical options for treating *WWOX*-related disorders.

## 1 Introduction

Developmental and epileptic encephalopathy-28, also known as *WWOX*-related epileptic encephalopathy (WOREE) syndrome, is an autosomal recessive disorder. It usually presents as refractory epilepsy and in severe cases can lead to early death (1).

The *WWOX* gene (OMIM:605131) is located at 16q23.1-q23.2, and it encodes an oxidoreductase enzyme with two WW domains. This gene resides at the fragile site FRA16D on chromosome 16q23 (2). Fragile sites are prone to genomic instability during DNA replication, making them susceptible to translocation and deletion. *WWOX* is a tumor suppressor gene implicated in various types of cancers, such as lung, esophageal and breast cancers (3). Recent evidence has revealed that mutations in *WWOX* are associated with epilepsy, with variant types including missense variants, nonsense variants, frameshift variants, duplications, and splice site mutations. Most of these mutations are compound heterozygous or homozygous, leading to WOREE syndrome (1, 4).

In this study, we conducted a molecular analysis of a patient with childhood epileptic encephalopathy from Lanzhou, China. We identified a novel homozygous mutation in the *WWOX* gene that had not been previously reported.

## 2 Case presentation

This study conforms to the case report (CARE) checklist consensus guidelines (Supplementary Material). This study was approved by the Ethics Committee of the Second Hospital of Lanzhou University. Written informed consent was obtained from the parents of this patient.

### 2.1 Clinical manifestation

The proband experienced an onset of symptoms at 1 month of age. His symptoms included staring into space, an asymmetrical mouth, and stiff limbs, which last approximately 30 s and resolved spontaneously.

The child was born without abnormalities. Both parents are healthy relatives (cousins). They had no family history of epilepsy, developmental disorders or other disorders.

**Figure 1:**
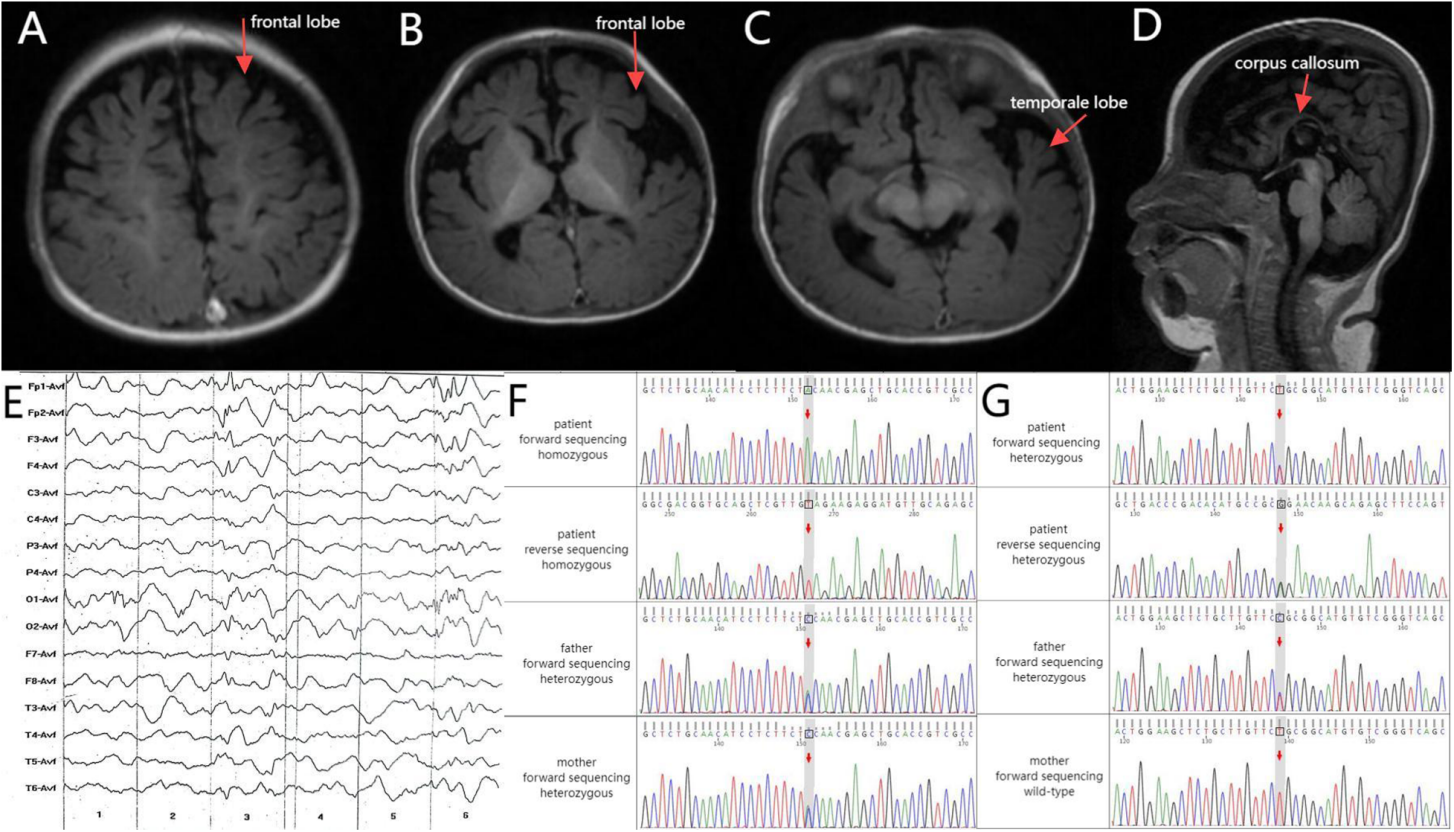
**(A–C)** Axial T1 brain MRI revealed deepened bilateral frontal sulci and cerebral fissures along with small gyri. **(D)** Sagittal T1 brain MRI revealed a slender corpus callosum. **(E)** Electroencephalography (4 months old): Increased medium-to-high amplitude spikes, as well as slow and sharp slow waves, were primarily observed in the bilateral frontal Poles and frontal and central regions during sleep. Asynchronized activity between the left and right sides was noted, occasionally spreading to the occipital areas, temporal areas, or adjacent leads. Schematic of the validation results by Sanger sequencing for the **(F)** *WWOX* and **(G)** *CACNA1A* variants. Upper panel: the proband. Middle panel: the proband's father. Lower panel: the proband's mother.

The child exhibited good physical development but some concerning symptoms. His eyes appeared glazed and unresponsive, lacking the ability to track objects. Additionally, his head was not well aligned vertically, and he could not turn over. His phenobarbital blood concentration was 26.2 μg/ml. Audiological testing indicated bilateral hearing loss and an absence of response to sound stimulation. Cardiac ultrasound revealed a patent foramen ovale and a left-to-right shunt at the atrial level. Brain magnetic resonance imaging (MRI) shows delayed neurological development of the child (Figures 1A–D). Representative electroencephalography at 4 months of age (Figure 1E). The clinical presentation and neuroimaging scans suggested epileptic encephalopathy. Therefore, the patient underwent genetic testing.

### 2.2 Genetic analysis

*WWOX* c.991C>A and *CACNA1A* c.4646A>G are categorized as having “unclear clinical significance”. A genetic test was conducted using the Illumina Nextseq 500 sequencing platform, with an average sequencing depth of ≥90× and coverage of 98% of intervals greater than 20×. Sanger sequencing was used to validate the test results. Various established computer algorithms were used to predict the conservation, pathogenicity, and harmfulness of the variants. The variant classification guidelines proposed by the American College of Medical Genetics and Genomics (ACMG) were used for variant classification. The whole-genome sequencing results revealed the presence of *WWOX* c.991C>A and *CACNA1A* c.4646A>G mutations in the peripheral blood DNA of the patient (Figures 1F,G). A mutation at base 991 in the DNA-coding region of the *WWOX* gene, resulting in a change from C to A, resulted in a mutation of amino acid 304 from glycine to serine (NM_016373.4: c.991C>A, p. Ser 304Tyr; Table 1). Another gene, *CACNA1A*, has a mutation of base 4,646 in the DNA-coding region from A to G, resulting in a change of amino acid 1,549 from glutamine to arginine (NM_001127222.2: c.4646A>G, p. Gln 1549Arg; Table 1).

**Table 1:** The *WWOX* gene variant c.991C>A and the *CACNA1A* gene variant c.4646A>G.

Gene	Chromosome location	Genetic variant	Zygote type	Disease name	Inheritance	Source of variation	Classification of variants
*WWOX*	Chr16:78466504	NM_016373.4: c.911C>A (p.Ser304Tyr)	Homozygote	Developmental and epileptic encephalopathy-28	AR	Paternal/maternal	Unclear clinical significance
*CACNA1A*	Chr19:13366018	NM_001127222.2: c.4646A>G (p. Gln 1549Arg)	Heterozygote	Developmental and epileptic encephalopathy-42	AD	Father	Unclear clinical significance

The ACMG recommends reporting pathogenic variants and suspected pathogenic variants with zygote types consistent with the inheritance pattern of related diseases in 78 monogenic disease-related genes. Both mutations are classified as clinically unknown*.* Regarding the *WWOX* gene, both parents of the patient tested positive for heterozygous variants [c.991C>A]. This indicates that the child's parents are recessive gene carriers and highlights the autosomal recessive inheritance pattern of the disease. Parental source analysis illustrated that the heterozygous mutation was inherited from both parents. The positive-sense strand of the *CACNA1A* gene in this patient is consistent with the wild type, whereas the antisense strand should have been A, but due to the mutation it was changed from A to G. In other words, it is c.4646A>G. According to Figure 1G, this mutation is of paternal origin. Furthermore, these mutations were identified as a novel mutation that has not been reported previously. They are not included in general population frequency databases, such as ClinVar, OMIM, HGMD, and gnomAD, as well as variant databases and population large-scale sequencing databases, indicating its rarity.

### 2.3 Treatment and follow-up

The patient was treated with 15 mg of phenobarbital twice daily and 1 ml of levetiracetam twice daily. The child was treated and discharged in good condition. But he died at the age of 6 months.

## 3 Discussion

WOREE syndrome was first reported by Abdel-Salam in 2014 (5), and roughly more than 90 cases have been reported to date. It is characterized by progressive psychomotor retardation, intractable epilepsy, spasticity, hyperreflexia, and electroencephalogram abnormalities. In some cases, there may be thinning of the corpus callosum, delayed myelination, cerebral atrophy, and eye abnormalities. The etiology of the disease is complex, and genetic testing studies have found that nearly one-third of children with developmental and epileptic encephalopathy have an inherited etiology, such as a new mutation in *WWOX* (NM_016373.4):c.516 + 1G>A originating from the father resulting in seizures, severe motor and mental retardation, and hypoplasia of the corpus callosum as found on brain nuclear magnetic resonance (6). Therefore, genetic testing plays an important role in identifying the cause of epileptic encephalopathy, guiding treatment and improving prognosis. Our patient presented with psychomotor retardation, intractable and refractory epilepsy, abnormal electroencephalogram, thinning of the corpus callosum, and impaired hearing development. Whole-exome testing revealed a missense mutation in the c.911c>(p. Ser304Tyr) gene of the affected child's *WWOX* (NM_016373.4) gene, originating from both related parents (cousins). Previous studies have suggested that mutations in the *WWOX* gene are strongly associated with autosomal recessive spinal cerebellar ataxia 12, sexually differentiated disorders, and susceptibility to seizures (7). However, the mechanism by which *WWOX* gene variants cause epileptic encephalopathy is unknown. *WWOX* is highly expressed in cortical neurons and hippocampus. It plays a key role in neuronal development, differentiation and protection. Its loss of function can lead to abnormal neuronal excitation, neuronal damage and neurodegeneration (8). It also involves mitochondrial dysfunction and apoptosis, which in turn leads to brain atrophy and volume loss (9).

In addition, mutations in the *CACNA1A* gene can lead to developmental and epileptic encephalopathy-42, which is characterized by various types of refractory seizures usually occurring in the first hours or days of life, with general developmental delay, mental retardation, with or without axial hypotonia, hyperreflexia, tremor, and ataxia (10). The main clinical features of the disease in question do not correspond well to the main clinical manifestations of the person examined. And it is an autosomal dominant disorder. Although our patient's *CACNA1A* gene originated from his father, the father did not show a similar phenotype. It is also according to the recommendations of the American College of Medical Genetics (ACMG), this type of genetic disease is likely to be unrelated to the prenatal clinical manifestations and diagnosis of the subject (11). Therefore, due to the heterozygosity of *CACNA1A* gene mutation, the mutation is less likely contributing to the clinical phenotypes.

Clinically, infantile epilepsy also needs to be differentiated from trauma, pyridoxal phosphate-dependent epilepsy, and neurologic damage caused by vitamin B12 deficiency. The current treatment is to control seizures and improve long-term neurodevelopment. The drug of choice is phenobarbital, which can also be used in combination with other drugs, including levetiracetam, sodium valproate, carbamazepine, midazolam, and lidocaine. Levetiracetam most effectively augments the anticonvulsant efficacy of phenobarbital, and fewer adverse effects can be expected in combination therapy because lower doses of antiepileptic drugs can be use (12). However, the combination may increase the risk of specific side effects, such as liver toxicity and rash. And drug resistance may develop. The mechanism of action of *WWOX* in the nervous system is unclear, and at this stage it is to control seizures in order to improve long-term prognosis. Meanwhile, gene therapy is promising and is currently under investigation (13). Neonatal gene therapy to restore *WWOX* expression using an adenoviral vector carrying the *WWOX* cDNA in the Mahertz and Akhilan laboratories reduced premature mortality and phonogenic epilepsy susceptibility and promoted partial normalization of development in *WWOX* mutant mice (14). This therapy is currently under approval. This new mutant locus expands the scope of gene therapy. In this case, the children's epileptic symptoms were poorly controlled after phenobarbital treatment and then improved slightly after levetiracetam treatment. Follow-up death occurred at 6 months of age.

## 4 Conclusion

The etiology of *WWOX*-associated epileptic encephalopathy is complex and more research is needed to further guide treatment and improve long-term prognosis. Moreover, this case confirms the involvement of *WWOX* in the pathogenesis of epileptic encephalopathy and supports the association between this mutation site and clinical phenotype. The pathogenicity of *WWOX* c.991C>A (p.Ser304Tyr) and its correlation with the clinical phenotype require validation through animal experiments and additional cases.

## Data Availability

The data presented in the study are deposited in the Figshare repository, the data can be found here: https://doi.org/10.6084/m9.figshare.26963461.v3.
